# Muscle oxygenation during dynamic plantar flexion exercise: combining BOLD MRI with traditional physiological measurements

**DOI:** 10.14814/phy2.13004

**Published:** 2016-10-24

**Authors:** Matthew D. Muller, Zhijun Li, Christopher T. Sica, J. Carter Luck, Zhaohui Gao, Cheryl A. Blaha, Aimee E. Cauffman, Amanda J. Ross, Nathan J.R. Winkler, Michael D. Herr, Kristen Brandt, Jianli Wang, David C. Gallagher, Prasanna Karunanayaka, Jeffrey Vesek, Urs A. Leuenberger, Qing X. Yang, Lawrence I. Sinoway

**Affiliations:** ^1^ Penn State Hershey Heart and Vascular InstitutePennsylvania State University College of MedicineHersheyPennsylvania; ^2^ Department of RadiologyCenter for NMR ResearchPennsylvania State University College of MedicineHersheyPennsylvania

**Keywords:** Blood pressure, heart rate, sympathetic nervous system

## Abstract

Blood‐oxygen‐level‐dependent magnetic resonance imaging (BOLD MRI) has the potential to quantify skeletal muscle oxygenation with high temporal and high spatial resolution. The purpose of this study was to characterize skeletal muscle BOLD responses during steady‐state plantar flexion exercise (i.e., during the brief rest periods between muscle contraction). We used three different imaging modalities (ultrasound of the popliteal artery, BOLD MRI, and near‐infrared spectroscopy [NIRS]) and two different exercise intensities (2 and 6 kg). Six healthy men underwent three separate protocols of dynamic plantar flexion exercise on separate days and acute physiological responses were measured. Ultrasound studies showed the percent change in popliteal velocity from baseline to the end of exercise was 151 ± 24% during 2 kg and 589 ± 145% during 6 kg. MRI studies showed an abrupt decrease in BOLD signal intensity at the onset of 2 kg exercise, indicating deoxygenation. The BOLD signal was further reduced during 6 kg exercise (compared to 2 kg) at 1 min (−4.3 ± 0.7 vs. −1.2 ± 0.4%, *P* < 0.001). Similarly, the change in the NIRS muscle oxygen saturation in the medial gastrocnemius was −11 ± 4% at 2 kg and −38 ± 11% with 6 kg (*P* = 0.041). In conclusion, we demonstrate that BOLD signal intensity decreases during plantar flexion and this effect is augmented at higher exercise workloads.

## Introduction

During exercise, skeletal muscle requires increased oxygen and nutrient delivery to meet the increased metabolism of contraction. In addition to increased blood flow, the skeletal muscle also has an impressive ability to increase oxygen extraction. Indeed, prior human experiments using cycle ergometry (Welch et al. 1977; Proctor et al. 2003a,b) and knee extension (Richardson et al. 1999; Amann et al. 2011) have shown that femoral blood flow increases and femoral venous oxygen saturation decreases during exercise, indicating not only improved oxygen delivery but also a larger arterial to venous oxygen difference. At submaximal intensities, both flow and oxygen extraction increase as workload increases (Proctor et al. 2003a,b; Damon et al. 2007b; Amann et al. 2011; Green et al. 2011; Richardson et al. 2015). Many laboratories have deployed Doppler ultrasound and near‐infrared spectroscopic (NIRS) methods to better understand muscle blood flow regulation and oxygen delivery during exercise (Heinonen et al. 2015). An alternative, yet relatively underutilized, approach to quantify local physiological responses during exercise is to use blood‐oxygen‐level‐dependent magnetic resonance imaging (BOLD MRI) in different muscle groups. Because MRI is subject to motion artifact, the leg must be still during image acquisition and most prior MRI studies have only evaluated postexercise blood flow (Isbell et al. 2006, 2007; Pollak et al. 2012; Caterini et al. 2015) or have used short duration isometric dorsiflexion exercise (which elicits minimal changes in leg blood flow (Meyer et al. 2004; Damon et al. 2007a,b; Towse et al. 2011). In this report, we present a novel application of BOLD MRI to quantify muscle deoxygenation during single‐leg dynamic plantar flexion exercise (i.e., a long duration laboratory stimulus similar to upright walking that significantly increases leg blood flow).

BOLD MRI is a common technique in neuroimaging (Ogawa et al. 1990). However, its application to study oxygen transport in skeletal muscle has not been fully developed. Oxyhemoglobin is diamagnetic and deoxyhemoglobin is paramagnetic (Aschwanden et al. 2014). The ratio of deoxyhemoglobin to oxyhemoglobin in the microvasculature influences the magnetic susceptibility of muscle tissue which, in turn, affects the transverse relaxation rates T2 and T2* of the proton signal (Noseworthy et al. 2003; Aschwanden et al. 2014). Because the BOLD signal is largely dependent on changes in T2*, it is not surprising that the most common stimuli employed in research settings alter the deoxyhemoglobin to oxyhemoglobin ratio (i.e., inhalation of hypoxic or hyperoxic gas, reactive hyperemia following thigh cuff occlusion, and lower leg exercise such as dorsiflexion or plantar flexion) (Noseworthy et al. 2003; Jacobi et al. 2012; Partovi et al. 2012). Mechanistic studies have shown that the BOLD signal in skeletal muscle is predominantly an intravascular effect; the extravascular effect is thought to contribute less than 5% of the total BOLD signal (Seiyama et al. 1988; Kennan et al. 1997; Meyer et al. 2004; Sanchez et al. 2010; Towse et al. 2011). While many studies have quantified BOLD signal intensity in the brain, there are not as many experiments evaluating skeletal muscle BOLD signal intensity. Two very recent MRI studies using 7‐Tesla MRI showed that single‐leg dynamic plantar flexion led to a decrease in gastrocnemius, soleus, and tibialis anterior BOLD signal intensity (Schmid et al. 2014; Schewzow et al. 2015) although the authors only evaluated one exercise intensity and did not measure heart rate (HR) or blood pressure (BP) which can influence leg blood flow and oxygenation. This “negative BOLD effect” in muscle during exercise differs from many prior leg exercise studies (Meyer et al. 2004; Damon and Gore 2005; Damon et al. 2007b; Towse et al. 2011) and at first glance is inconsistent with prior neuroimaging studies that ascribe a positive BOLD effect to increases in blood flow (i.e., metabolic vasodilation in the brain) (Ogawa et al. 1990). It should be noted that the BOLD signal is influenced by blood flow, blood volume, and blood oxygenation (Meyer et al. 2004; Damon and Gore 2005; Damon et al. 2007b; Towse et al. 2011; Schmid et al. 2014; Caterini et al. 2015; Schewzow et al. 2015); very few publications have used multiple different techniques to examine the relative importance of these factors (Damon and Gore 2005; Towse et al. 2011). Additionally, the mode, duration, and intensity of exercise may impact the BOLD signal during plantar flexion. In order to implement BOLD MRI in physiological research, more work is needed to address these gaps in knowledge.

The purpose of this study was to characterize skeletal muscle BOLD responses during steady‐state plantar flexion exercise (i.e., during the brief rest periods between muscle contraction). Based on existing knowledge about blood flow and muscle oxygenation during exercise, we reasoned that if the muscle BOLD signal increases during exercise then the temporal responses at 2 and 6 kg workloads would be similar in magnitude to popliteal artery blood velocity responses (i.e., muscle blood flow is known to be higher at higher workloads). If, however, we observed a “negative BOLD effect” we expected the temporal responses at 2 and 6 kg workload to be more similar to the NIRS responses (i.e., muscle oxygen saturation is known to become more negative at higher workloads because oxygen extraction increases). Accordingly, we hypothesized that BOLD signal intensity is different during exercise at 2 kg versus exercise at 6 kg.

## Methods

### Ethical approval

These protocols were approved in advance by the Penn State College of Medicine Institutional Review Board. All procedures were conducted according to the guidelines set forth in the Declaration of Helsinki. All volunteers provided written informed consent prior to participation.

### Design and subjects

This study used repeated measures, within‐subjects design. Participants underwent different human physiology experiments on separate days. Physiological variables were measured over time (i.e., before, during, and after exercise) and differences between the 2 and 6 kg workloads were quantified. The experiments were not randomized or blinded.

All subjects were healthy men (*n* = 6, range 23–64 years) who were not taking any medications. They were normotensive, nonobese, and nonsmokers who were recreationally active but not competitive athletes. Their height (177 ± 3 cm), weight (80.6 ± 7.7 kg), and body mass index (25.6 ± 1.9) were within normal limits. The sample size was determined after the first four subjects had completed testing. Specifically, we determined that if the mean difference in the BOLD signal intensity in the medial gastrocnemius in the first minute of exercise (between 2 and 6 kg) was 3% with a standard deviation of 1.7%, we would need to enroll six subjects to be able to reject the null hypothesis with 90% power and a type 1 error of 0.05.

### Plantar flexion exercise model

**Figure 1:**
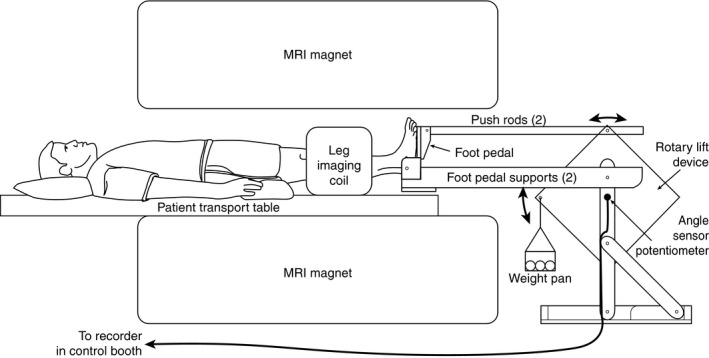
Magnetic resonance imaging (MRI) compatible leg exercise device. The subjects were positioned supine with the largest part of the lower leg in the coil. Sand bags were added to the weight pan to increase the workload. A personal computer in the control room was connected via an MRI compatible cord to monitor the range of motion. This same device was also used for ultrasound studies (Protocols 1 and 3).

All experiments were conducted in the supine posture in a thermoneutral clinical research laboratory (Protocols 1 and 3) or in a research MRI scanner (Protocol 2, Magnetom Trio; Siemens Medical, Erlangen, Germany). All experiments followed similar procedures. After 15 min of quiet rest, HR and BP were obtained. The foot was then secured to a wooden pedal that was connected via wooden pushrods to a wooden rotary lift device containing a plastic weight pan (Fig. 1). Using 0.5 kg sandbags, either 2 or 6 kg of weight was added to the weight pan. Based on the device, 2 kg is ~1.76 J per contraction and 6 kg is ~5.28 J per contraction. This device was used for both MRI and ultrasound studies and was constructed after consulting previous literature (Haseler et al. 1999, 2004, 2007; Isbell et al. 2007; Esterhammer et al. 2008; Ghomi et al. 2011; Green et al. 2011; Pollak et al. 2012; Donnelly and Green 2013). For each study, there was a 1‐min baseline followed by 14 min of exercise and 5 min of recovery. During the 14‐min exercise bout, subjects performed single‐leg plantar flexion exercise in between image acquisition (i.e., the leg was at rest with the foot at 90° relative to the fibula with minimal tension in the leg muscles when images were obtained). The duration of each MRI scan was 3000 ms, divided into 789 ms of image acquisition and 2211 ms of delay time during which the plantar flexion contraction occurred. There was no gradient noise during the delay time, which cued the subject to exercise. Thus, 20 contractions were performed each minute and 280 contractions were performed during each exercise bout. Practice trials (5–10 contractions) were conducted ~15 min prior to image acquisition to ensure the subjects could maintain the cadence. Two investigators (MDM and CAB) remained in the room during data collection and occasionally reminded the subjects to “exercise in between the sound and be completely still during the sound.” A potentiometer attached to the shaft of the rotary left device sensed the angle of rotation and the signal was collected into PowerLab (ADInstruments, New South Wales, Australia). Additionally, a timing pulse coincident with each imaging interval was recorded.

### Protocol 1: popliteal artery velocity responses

Protocol 1 was conducted in the Clinical Research Center of the Clinical and Translational Science Institute at Penn State College of Medicine. Brachial artery BP was measured at baseline with an oscillometric device (Philips SureSigns VS3, Andover, MA). Heart rate was measured by 3‐lead EKG (Cardiocap/5, Madison, WI), beat‐by‐beat BP was measured by photoplethysmography (Finometer, FMS, Arnhem, The Netherlands), and respiratory movement was monitored with a pneumobelt. Popliteal artery time average peak velocity was measured with a Philips iE33 (Bothell, WA) similar to previously published methods (Richardson et al. 2001; Green et al. 2011; Layec et al. 2014; Hart et al. 2015) and was logged into PowerLab, using a custom Doppler audio converter (Herr et al. 2010). All physiological variables were collected continuously during baseline, 14‐min of exercise (2 or 6 kg), and recovery. Ratings of perceived exertion and leg pain were determined at the end of the exercise (Borg 1982). The 2 kg workload was performed first followed by a 20‐min rest period and then the 6 kg workload was performed with the same leg. For technical reasons, in some subjects, the 6 kg workload was performed on a separate day.

### Protocol 2: functional MRI of the lower leg

Protocol 2 was conducted in the Center for NMR Research at Penn State College of Medicine. Procedures were similar to Protocol 1 except that beat‐by‐beat HR and BP were not obtained. Rather HR was monitored with a pulse oximeter on the finger and BP was measured every 2 min by an MRI compatible device (Precess MRI Compatibility; Invivo Corperation, Gainesville, FL). In general, the 2 kg workload and 6 kg workloads were performed on separate days but one subject performed the 2 kg workload and the 6 kg workload on the same day separated by 20 min of rest. An eight‐channel knee coil was positioned around the largest part of the lower leg, and a T2*‐weighted echo planar imaging method was used, with the echo time = 25 ms, repetition time = 3000 ms, flip angle = 70 degrees, field of view = 160 × 160 mm, matrix size = 64 × 64, number of slices = 10, and slice thickness = 5 mm. A chemically selective saturation pulse was applied prior to each data acquisition to suppress the signal from fat. A single shot trajectory was employed during the EPI acquisition. As stated above, each MRI scan imaged for 789 ms followed by a delay of 2211 ms during which plantar flexion contraction occurred. All 400 scans (20 scans per minute × 20 min) were analyzed offline (please see data processing below).

### Protocol 3: popliteal artery diameter and NIRS

Based on the data obtained in Protocols 1 and 2, five of the six subjects returned to the Clinical Research Center for additional testing. These studies were similar to Protocol 1 except that ultrasound was used to measure popliteal artery diameter instead of velocity. The 2 kg workload was performed first followed by a 20‐min rest period and then the 6 kg workload was performed with the same leg.

Additionally during these studies, NIRS muscle oxygen saturation (oxyhemoglobin/total hemoglobin, Moxy muscle oxygen monitor; Fortiori Design LLC, Hutchinson, MN) was quantified continuously over the medial gastrocnemius of the exercising leg, similar to previous plantar flexion studies (Layec et al. 2014; Hart et al. 2015). The Moxy NIRS device uses an algorithm based on Monte Carlo modeling to trace the propagation of light through tissue and the Beer–Lambert Law to distinguish between known optical properties for the given chromophores oxygenated hemoglobin and deoxygenated hemoglobin. This NIRS device is capable of isolating a signal to the skeletal muscle, using this complex algorithm that accounts for the light absorbing properties of four mediums (epidermis, dermis, adipose tissue and skeletal muscle) at various thicknesses. The algorithm is specifically designed to filter out signals from skin blood flow, changes in temperature, and pulsatile signals. NIRS uses a combination of one light emitting diode and two 1.5 mm detector diodes located at 11.75–13.25 and 24.25–25.75 mm to detect near‐infrared light wavelengths (*λ*) between 630 and 850 nm. Provided that adipose tissue thickness is no greater than 12.5 mm, the monitor is capable of measuring the oxygen saturation of skeletal muscle. Based on our pilot studies conducted in other subjects where adipose tissue thickness was measured using ultrasound (range 4.1–9.5 mm), we believe all subjects were within the acceptable range for the device to make accurate recordings.

### Data processing

Beat‐by‐beat data for Protocols 1 and 3 were collected at 200 Hz (16 channel PowerLab; ADInstruments) and analyzed offline, using LabChart (ADInstruments). We separated each minute into three 20‐sec bins to evaluate temporal responses and the mean values for each bin were recorded. Popliteal images were analyzed on the iE33 ultrasound and the time average peak values from the resting phase (between muscle contractions) were recorded. Because popliteal artery measurements were always obtained in the last 20 sec of each minute, data from this bin are presented. We decided not to calculate popliteal blood flow for two reasons. First, diameter and velocity were obtained on separate days. Second, during calf muscle contraction, it is not possible to measure the velocity in this artery even though some flow likely occurred. Because NIRS, HR, and BP signals are of equal magnitude during both rest and contraction phases of exercise, these data are reported for the entire 20 sec bin.

For Protocol 2, HR and BP were logged into PowerLab every 2 min. MRI data were analyzed offline as follows. First, realignment, slice timing correction, and motion correction were performed with Statistical Parametric Mapping version 8 (SPM8, Wellcome Trust Center for Neuroimaging, University College London, London, UK). Next, region of interest selection within each of the four muscle groups was performed with MRIcron (CRNL, University of South Carolina, Columbia, SC). The regions of interest were exported back to SPM8 for extraction of a BOLD signal time course from each muscle group. The BOLD signal time course was computed from the mean intensity value within the ROI at each of the 400 time points. A wavelet decomposition was then performed using Matlab's Wavelet Toolbox (Mathworks, Natick, MA) with the “*bior4.4*” basis function and five levels, followed by reconstruction of the approximation coefficients. The third level approximation (example Fig. 4D) was found to smooth spikes in the data while retaining the overall shape of the BOLD time course, and was used for all subjects. Using Excel, percent changes from baseline in BOLD signal intensity were calculated for the medial gastrocnemius, lateral gastrocnemius, soleus, and tibialis anterior consistent with prior publications (Wu et al. 2008; Schewzow et al. 2015). As stated above, the primary outcome was the percent changes in the medial gastrocnemius BOLD signal intensity in response to steady‐state exercise.

### Statistical analysis

Statistics were conducted using SPSS 21.0 (IBM Corp., Armonk, NY) and graphics were produced with Microsoft Excel and Adobe Creative Cloud. Because the overall study used a repeated measures design with exercise workload (2 kg vs. 6 kg) as a within subjects factor, repeated measures ANOVA was conducted on the raw physiological values whenever possible. However, some measurements were only obtained at intermittent time points (HR and BP during Protocol 2) or were evaluated qualitatively (comparing different muscle groups) or were calculated as percent change from baseline (BOLD and NIRS data). For Protocol 1, repeated measures ANOVA was used to determine the effect of exercise workload on HR, MAP, and popliteal velocity over time. Planned paired *t*‐tests were conducted at 1, 2, 3, and 4 min of exercise (using the last 20 sec of each minute) based on our previous publications with single‐leg plantar flexion exercise (Muller et al. 2012, 2015; Drew et al. 2013). For Protocol 2, mean data from all 400 scans were plotted and paired *t*‐tests were conducted at 1, 2, 3, and 4 min of exercise. In order to account for multiple comparisons, we used the Holm–Bonferroni step‐down adjustment. For Protocol 3, percent changes in popliteal diameter and NIRS at the end of exercise were compared with paired samples *t*‐test. For all protocols, ratings of perceived exertion and leg pain between workloads were compared with Wilcoxon nonparametric tests. Data are shown as mean ± SEM unless otherwise stated and *P* values < 0.05 were considered statistically significant.

## Results

### Protocol 1: popliteal artery velocity responses

**Figure 2:**
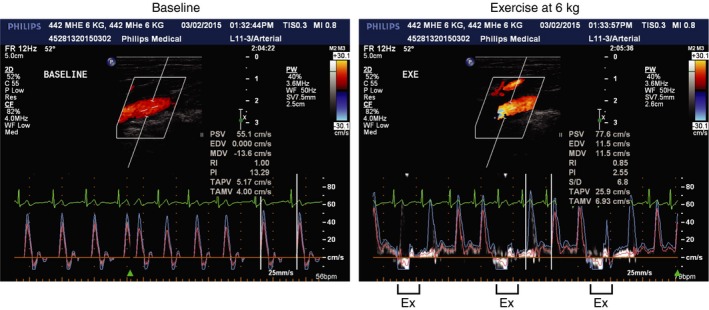
Original Doppler ultrasound recordings of popliteal blood flow velocity recorded at baseline (left panel) and during single‐leg dynamic plantar flexion exercise at 6 kg (right panel) in a 62‐year‐old healthy man. Note that in the left panel, velocity waveforms are triphasic whereas in the right panel the velocity waveforms are not triphasic and can only be measured in between exercise (Ex) contractions.

**Figure 3:**
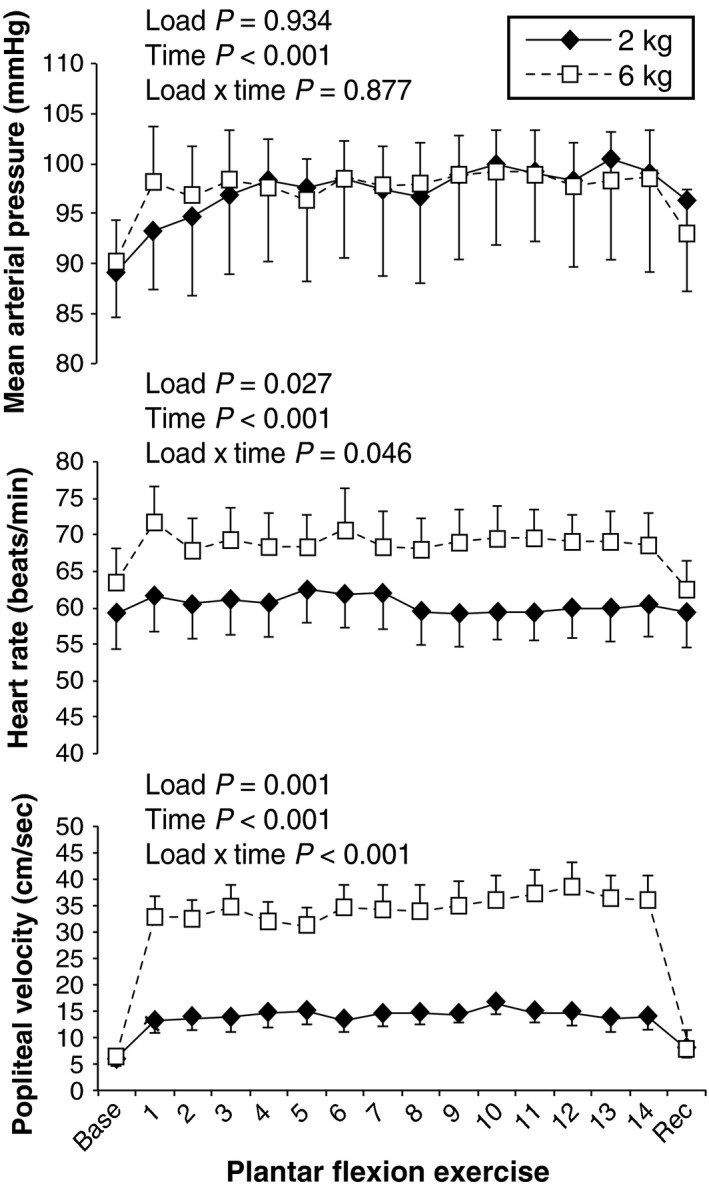
Systemic hemodynamic responses to single‐leg dynamic plantar flexion exercise at 2 kg (black diamonds) and 6 kg (white squares) in Experiment 1. All parameters significantly increased over time. The magnitude of difference between workloads was most noticeable for popliteal mean velocity (bottom panel), which was consistent with the purpose of our study (i.e., influencing local blood flow regulation while not significantly raising sympathetic tone). Data are M ± SEM.

An example recording of popliteal velocity during the 6 kg workload is shown in Figure 2 and mean data between workloads are shown in Figure 3. As expected, plantar flexion at both 2 and 6 kg increased MAP, HR, and popliteal mean velocity over time (main effect *P* < 0.001, Fig. 3). HR demonstrated a load × time interaction and HR was higher during 6 kg regardless of time but none of the individual comparisons reached statistical significance. The change in popliteal mean velocity was markedly enhanced at 6 kg versus 2 kg at all time points (*P* < 0.001). The percent change in popliteal blood flow velocity from baseline to the end of exercise was 151 ± 24% during 2 kg and 589 ± 145% during 6 kg. Neither of these workloads was taxing. Indeed, the median rating of perceived exertion was 9.5 (“very light”) following 2 kg and 11 (“light”) following 6 kg (*P* = 0.033). No leg pain was experienced.

### Protocol 2: functional MRI of the lower leg

**Figure 4:**
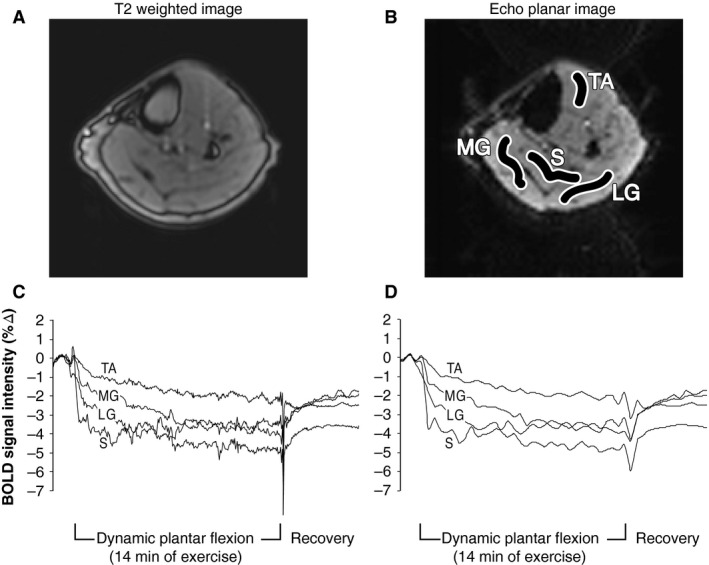
Magnetic resonance imaging of the lower leg from one subject (same individual as Fig. 2). (A) T2‐weighted image to orient the reader. (B) Echo planar image with regions of interest that was used for analysis: tibialis anterior (TA), soleus (S), medial gastrocnemius (MG), and lateral gastrocnemius (LG). (C) Unfiltered blood‐oxygen‐level‐dependent (BOLD) signal intensity data during 6 kg exercise shown as a percent change from baseline. (D) The same BOLD signal intensity data after wavelet‐based filtering.

**Figure 5:**
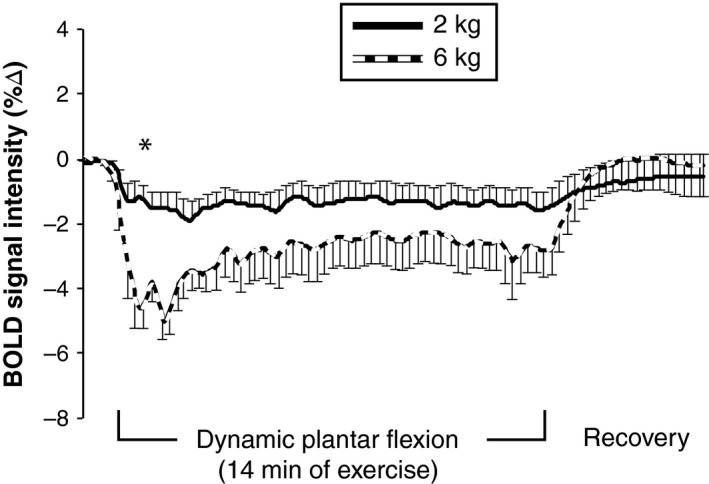
Time‐course of blood‐oxygen‐level‐dependent (BOLD) signal intensity in the medial gastrocnemius in response to plantar flexion at 2 kg (solid line) and 6 kg (dashed line). All 400 data points from the 20 min scan are presented, but only some of the error bars are shown to improve clarity. A greater negative BOLD response was found during exercise with 6 kg, indicating lower deoxyhemoglobin concentration. Data are M ± SEM. Planned comparisons were conducted between 2 and 6 kg at specific time points (please see the text for details). *indicates significant difference between workloads (*P* < 0.05).

An example of each individual muscle response to 6 kg is shown in Figure 4. Mean data from the medial gastrocnemius (Fig. 5) demonstrate that there was an abrupt decrease in BOLD signal intensity at the onset of exercise. This reduction in the BOLD signal was augmented during 6 kg exercise (compared to 2 kg) at 1 min (−4.3 ± 0.7 vs. −1.2 ± 0.4%, *P* < 0.001). Because of the Holm–Bonferroni step‐down adjustment, we did not observe statistical significance at 2 min (−4.0 ± 0.8 vs. −1.5 ± 0.6%, *P* = 0.08) or 3 min (−3.5 ± 0.6 vs. −1.4 ± 0.4%, *P* = 0.092). Had we not corrected for multiple comparisons, nearly all time points during exercise would have been significantly different between treatments.

The increase in MAP (5 ± 1 mmHg) and HR (2 ± 1 bpm) at the end of the 2 kg exercise tended to be lower than the responses to 6 kg (10 ± 2 mmHg and 6 ± 2 bpm, respectively) but these comparisons did not reach statistical significance (*P* both > 0.2). The median rating of perceived exertion was 9 (“very light”) following 2 kg and 12 (between “light” and “somewhat hard”) following 6 kg (*P* = 0.013).

### Protocol 3: popliteal artery diameter and NIRS

Similar to Protocol 1, plantar flexion at both 2 and 6 kg elicited increases in MAP and HR over time but the magnitude was similar for both workloads. Neither 2 kg (baseline 0.622 ± 0.028 cm to end of exercise 0.630 ± 0.026 cm, *P* = 0.473) nor 6 kg (baseline 0.626 ± 0.028 cm to end of exercise 0.625 ± 0.024 cm, *P* = 0.792) had an effect on popliteal artery diameter.

**Figure 6:**
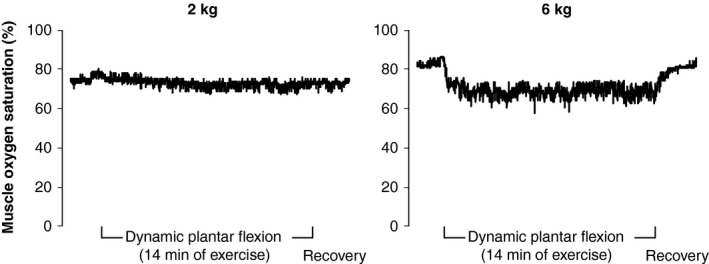
Individual recordings of muscle oxygen saturation obtained from near‐infrared spectroscopy during 2 and 6 kg plantar flexion. These data were obtained from the same individual shown in Figures 2 and 4.

Figure 6 includes examples of NIRS muscle oxygen saturation from one subject. Considering the entire group of subjects, the percent change in the NIRS muscle oxygen saturation (baseline to end of exercise) in the medial gastrocnemius was −11 ± 4% at 2 kg and −38 ± 11% with 6 kg (*P* = 0.041). The median rating of perceived exertion was 7 (“extremely light”) following 2 kg and 11 (“light”) following 6 kg (*P* = 0.021). Taken together, these data suggest that our exercise paradigm had a significant effect on muscle oxygenation (i.e., reduced venous oxygen saturation at 6 kg).

## Discussion

Recent review articles have suggested that BOLD MRI is a useful tool to study human physiology and pathophysiology (Partovi et al. 2012; Aschwanden et al. 2014). However, before BOLD MRI can be implemented as a diagnostic or prognostic tool in skeletal muscle, more rigorous physiology experiments are needed to understand the BOLD signal across a wide range of blood flow and oxygenation levels. This is especially true because, unlike in the brain, the BOLD signal is not a surrogate for blood flow in skeletal muscle (Towse et al. 2011). Considering this background, the purpose of this study was to characterize skeletal muscle BOLD responses during steady‐state plantar flexion exercise (i.e., during the brief rest periods between muscle contraction). By design, these studies were intended to provide a low workload as might be seen with slow walking and thus elicit minimal changes in HR and BP (i.e., to evaluate local physiological process relevant to activities of daily living in vivo). In this report, we not only present methodological advances but also the novel finding that BOLD signal intensity is lower during exercise at 6 kg compared to exercise at 2 kg.

Figures 1 and 2 indicate that our exercise paradigm is technically feasible in both the MRI environment and also in vascular ultrasound laboratories. Moreover, the popliteal blood flow velocity and diameter data we obtained between muscle contractions is consistent with a recent plantar flexion study performed by Green et al. (2011). As expected, steady state popliteal blood flow velocity responses were three times greater during 6 kg plantar flexion compared to 2 kg (Fig. 3). The difference between workloads was manifest within the first minute of exercise.

Considering the BOLD data (Figs. 4 and 5), the onset of exercise elicited a rapid decrease in BOLD signal intensity within the first few seconds of both 2 and 6 kg exercise. At 1 min of exercise, the reduction in BOLD signal intensity was significantly different between workloads. Indeed, steady‐state BOLD signal intensity in the medial gastrocnemius decreased twice as much during 6 kg compared to 2 kg (Fig. 5). Steady state NIRS muscle oxygen saturation also decreased two‐to three‐times more with 6 kg than with 2 kg. Stated another way, our NIRS data are directionally similar to the BOLD data whereas the popliteal ultrasound data are opposite to BOLD. Based on these observations, we speculate that skeletal muscle BOLD responses in healthy humans are primarily due to deoxygenation of active muscle (i.e., in healthy humans, the BOLD response primarily reflects a decrease in venous oxygen saturation and not an increase in leg blood flow). However, we cannot prove cause and effect with the current data. To our knowledge, we are the first to use vascular ultrasound, BOLD MRI, and NIRS to study the acute effects of plantar flexion exercise (i.e., during the brief rest periods between muscle contraction). Had we not used these three methodologies in concert, we would not be able to assert that the negative BOLD is due to deoxygenation of active muscle. For this reason, the negative BOLD that we observed is most convincing when viewing Protocols 1–3 in aggregate.

One of the advantages of BOLD MRI over other imaging modalities is the high spatial resolution. Indeed, we demonstrate that the BOLD signal intensity decreased in all muscle groups measured (Fig. 4). This suggests that activities that engage all muscles of the lower leg (e.g., walking) likely elicit deoxygenation of hemoglobin. Similar decreases in all muscle groups were observed in a recent plantar flexion study by Schewzow et al. (2015). A study by Behnke et al. (2003) performed in rodents demonstrated that microvascular oxygen kinetics were different between the fast‐twitch peroneal muscle and slow twitch soleus muscle during the transition from rest to steady‐state exercise. Based on the literature (Layec et al. 2014; Hart et al. 2015), we chose to focus on the medial gastrocnemius (Fig. 5) in this study, but future studies may investigate the oxygen kinetics and recruitment strategies of muscle groups of varying fiber types. In general, our data support prior to treadmill walking studies that used NIRS to quantify calf muscle deoxygenation (Kenjale et al. 2011; Gardner et al. 2014). The use of NIRS may be advantageous to study muscle blood flow regulation because this technology is operable during both rest and contraction phases; BOLD MRI requires the leg to momentarily be at rest. However, because NIRS lacks spatial resolution and is also influenced by changes in skin temperature (Davis et al. 2006), we believe the use of BOLD MRI during leg exercise is an important methodological advancement to this field.

There are several potential contributors to the observed BOLD signal changes. One possible factor is exercise‐mediated T2 changes, which are primarily associated with increased rates of metabolism. Such changes are brought about by increases in intracellular free water content and acidification. Free water changes are related to the intracellular accumulation of osmolytes, which pull water into the cell through osmotic pressure gradients (Prior et al. 2001; Damon et al. 2002). Acidification results from the accumulation of glycolytic intermediate and end‐products produced through anaerobic glycolysis (Fung and Puon 1981; Damon et al. 2002). The level of exercise in our study was rated as light by the participants, no leg pain was experienced, and the contractions were about 2 sec in duration. Given the workload, the exercise is most likely categorized as aerobic. The exercise‐induced pH and intracellular water changes required to alter T2 typically result from the accumulation of end‐products of anaerobic metabolism, which should not be the case here (Damon et al. 2002; Lanza et al. 2005; Forbes et al. 2009). Thus, T2 changes are not expected to be a factor in our experiments.

Another potential contribution could be due to the inflow effect. The inflow effect occurs when blood flowing into the imaging plane possesses full longitudinal magnetization, instead of the expected partial magnetization that depends upon TR and tissue T1. Prior studies have examined the contribution of the inflow effect and found it to be minimal (Meyer et al. 2004; Damon et al. 2007a). Inflow effects are not expected to be significant given the scan parameters used in our study. Assuming a tissue T1 of 1200 ms, blood T1 of 1500 ms, and blood volume of 3%, and the scan parameters TR = 3000 ms and flip angle = 70 degrees, fully relaxed blood entering the slice would only increase the voxel signal 0.29% relative to blood in the steady state. This behavior is mainly due to the high ratio of TR to T1.

Prior leg imaging studies suggest that in healthy muscle the BOLD response is almost purely intravascular (Meyer et al. 2004; Wigmore et al. 2004; Sanchez et al. 2010; Towse et al. 2011) which makes any extravascular BOLD effect very minimal in our study. The extravascular BOLD response refers to the dephasing of spins within magnetic field gradients immediately surrounding the capillaries, and depends upon factors such as capillary density, orientation, and radius, the diffusion coefficient of water perpendicular to the capillary axis, and magnetic field strength (Stables et al. 1998). There is also a dependence upon relative blood volume, fractional oxyhemoglobin saturation, and hematocrit. The near parallel orientation of the capillaries in the leg relative to the main field produces a condition where there is no additional magnetic field gradient surrounding the capillary (Meyer et al. 2004; Wigmore et al. 2004; Sanchez et al. 2010; Towse et al. 2011).

The observed signal changes in our study can be explained in terms of the intravascular BOLD response. The intravascular BOLD response is modeled in terms of the T2* and relative volume of blood and muscle compartments within a voxel (Towse et al. 2011). The T2* further depends upon the fractional oxyhemoglobin saturation and hematocrit. Alterations in blood flow can effect local changes in blood volume and subsequently influence the BOLD response. In the presence of significant alterations in blood flow, separation of the T2* and blood volume contributions is not straightforward (Duteil et al. 2006). In this study, we are uncertain if blood pooling occurred due to the coil. Thus, we cannot completely exclude the effect of blood volume in this study. In all our studies, HR and MAP responses were small and the exercise was rated as “extremely light” to “light” so it is unlikely our data are confounded by changes in systemic hemodynamics.

Supplementing the BOLD data with additional MRI measurements is one way of addressing this issue. Arterial Spin Labeling techniques can offer useful information about local perfusion and blood flow, which can potentially aid interpretation of the BOLD signal (Pollak et al. 2012; Grozinger et al. 2014; Schewzow et al. 2015). The PIVOT technique measures perfusion, T2*, and oxygen saturation of blood in the larger arteries or veins (Englund et al. 2013). Relative changes in T2* of muscle voxels are due to changes in capillary and venule blood T2* only due to the lack of an extravascular BOLD response. The oxygen saturation of the large draining veins (peroneal, posterior tibial, anterior tibial) may give insight into the oxygen consumption of the respective muscle group served by each vein.

In conclusion, our studies present a promising methodological approach to evaluate skeletal muscle oxygenation during exercise. We demonstrate that BOLD signal intensity can be measured simultaneously in several muscle groups during exercise (i.e., during the brief rest periods between muscle contraction) and future studies may evaluate this process in patients with cardiovascular disease.

## Conflict of Interest

None declared.
